# The Conceptualization of Space: *Places* in Signed Language Discourse

**DOI:** 10.3389/fpsyg.2020.01406

**Published:** 2020-07-08

**Authors:** Sherman Wilcox, Rocío Martínez

**Affiliations:** ^1^Department of Linguistics, University of New Mexico, Albuquerque, NM, United States; ^2^Instituto de Lingüística, Facultad de Filosofía y Letras, Universidad de Buenos Aires and Consejo Nacional de Investigaciones Científicas y Técnicas (CONICET), Buenos Aires, Argentina

**Keywords:** signed language, cognitive grammar, pointing, reference points, deixis

## Abstract

We examine the conceptualization of space in signed language discourse within the theory of cognitive grammar. Adopting a Places view, we define Place as a symbolic structure that associates a schematic semantic pole and a schematic phonological pole. Places acquire full contextual meaning and a specific spatial location in the context of a usage event. In the present article, we analyze the referential function of Places in different grammatical constructions throughout a selection of videos produced by deaf Argentine Sign Language signers. Our analysis examines Places, which are associated with entities in the surrounding spatial environment as well as Places that are created or recruited in discourse without reference to surrounding physical entities. We observe that Places are used in pointing, placing, and other grammatical constructions in order to introduce and track referents in ongoing discourse. We also examine the use of conceptual reference points, by which Places afford mental access to new related concepts that are the intended focus of attention. These results allow us to discuss three related issues. First, for signed language discourse, space is both semantically and phonologically loaded. Signers’ semantic and phonological choices for Place symbolic structures are motivated by embodied experience and the abstraction of usage events. Second, Places occur along a continuum from deixis to anaphor, united by the same conceptual system and differing in extent of phonological subjectification. Third, we suggest developmental implications of our Place analysis.

## Introduction

Signed languages are uniquely suited for studying the conceptualization of space. Signs are produced by moving hands in three-dimensional space. As Stokoe (1980) observed, “In producing a sign language utterance, some part (or parts) of the signer’s body acts. If the active part is mobile enough, there are various places in which the action may occur, i.e., begin, take place, or end.” These three aspects of a signed utterance have been recognized as three basic phonological parameters: handshape (active part), location (place), and movement (action). Of the three, the most significant for revealing how signers conceptualize space is location. Signs were originally described as incorporating locations on or near the body and those that are produced in an unmarked three-dimensional signing space in front of the signer’s head and torso, extending from a little above the head to a little below the waist. It is signs that are produced in this signing space that we examine, and our primary focus is their referential function.

Research on reference in signed languages has been closely connected to the use of space. Based on the theoretical framework used and the similarities of their proposals, these studies can be grouped into four main views on the use of space for achieving reference in signed languages: (1) the referential locus (R-locus) view, (2) the mental spaces view, (3) the locus with semantic-pragmatic conventions view, and (4) the symbolic Places view.

Researchers working within formalist theory adopt a *referential locus (R-locus) view*, claiming that spatial locations are used for identifying referents previously associated with that location. These are called R-loci. R-loci are distinguished from referential indexes (R-indexes): The former are the physical spatial locations toward which a signer points, whereas the latter are abstract formal devices indicating reference within and across sentences (Lillo-Martin and Klima, 1990). Within this view, the location in space for achieving reference (R-locus) is randomly chosen by the signer. More recent research claims that, whereas abstract indices are part of the grammar, loci are determined outside of grammar. This leads some proponents of this view to a provocative conclusion: “On our view, the grammar doesn’t care which point in space is used for a particular referent. Abstract indices are part of the grammar, but loci are determined outside of grammar. Therefore, the connection between referents and loci requires language to interface with gesture” (Lillo-Martin and Meier, 2011, p. 121)^1^.

The *mental spaces view* is based on mental space theory (Fauconnier, 1985, 1997). As applied to signed languages, its main proponent is Liddell (1995). In his first approach, Liddell proposed that three mental spaces are recruited for creating and maintaining reference in American Sign Language (ASL) discourse: real space, surrogate space, and token space. Real space is a person’s current conceptualization of the immediate environment based on sensory input. Real space is used when the signer refers to entities that are conceptualized as being physically present, such as using a pronoun toward the addressee or toward objects that are present in the physical situation. Surrogate space describes a type of full-sized, invisible entity. Pronouns and indicating verbs make reference to a surrogate by being directed toward it. Tokens are entities that, like surrogates, are given manifestation in physical space. The difference is that, unlike surrogates, tokens use a limited size of the signing space in front of the signer and only assume third person roles in discourse. Liddell (2003) later revised this theory, following blending theory (Fauconnier and Turner, 1996), showing how real, surrogate, and token space become part of different blended mental spaces^2^.

Many sign linguists who adopt the mental spaces view claim that the number of locations in space is unlistable and, therefore, cannot be an element of the grammar. According to this claim, any specific instance of a pronoun directed toward an entity will be a combination of lexically fixed features encoding the symbolic pronoun and a non-symbolic pointing direction selected for the specific context in which it is being used (Liddell, 2003). In addition to pronouns, other structures receive similar treatment. For instance, it is claimed that directional verbs, which are called indicating verbs by Liddell, are composed of both lexically fixed features and gestural elements. The actual placement of the hand during the initial or final hold is said to be “gradient” because it depends on the locations of the entities toward which it is directed. Comparable analyses can also be found in research discussing language–gesture fusions (Fenlon et al., 2018).

The *locus with semantic-pragmatic conventions view* (Engberg-Pedersen, 1993) defines locus as an abstract category whose members are specific spatial loci in paradigmatic contrast. Engberg-Pedersen (1993, p. 69) asserts that conventions influence the signer’s choice of loci. The space around the signer is semantically “loaded”: The choice of a locus for a given referent is not arbitrary, but influenced by semantic and pragmatic conventions. For instance, the convention of semantic affinity states that referents with semantic affinity to each other (for example, a person and the place where she works or a person and his possessions) are usually represented by the same locus unless they need to be distinguished for discourse reasons; the convention of comparison occurs when a signer chooses the locus forward-sideward-left for one referent and the locus forward-sideward-right for another referent when she wants to compare or contrast the two referents. These conventions are neither exhaustive, nor do they have the character of obligatory rules.

We adopt a *Places view* (Wilcox and Occhino, 2016; Martínez and Wilcox, 2019), a usage-based approach developed within the model of cognitive grammar (CG; Langacker, 1987, 1991, 2008; Wilcox, 2014). Our view is grounded in sensory and physical experience and, thus, is an embodied approach in which embodied cognition and experiential conceptual archetypes are fundamental (Langacker, 2006; Barsalou, 2008). Within this approach, the unlistability and the gradience of the locations in signing space are not matters of concern given that we assume a non-structuralist conception of language and its units (Wilcox, 2014). The locations that signers use meaningfully within signing space, as well as any other unit, cannot be conceptualized *a priori* as discrete and categorical, but as elements that arise in a bottom-up fashion. In previous studies, we have called these meaningful locations in signed languages Places (Wilcox and Occhino, 2016; Martínez and Wilcox, 2019). A Place is a symbolic structure, a pairing of a meaning and a location in space that plays a major role in reference in signed languages. Places are, thus, semantically and phonologically substantive, derived from embodied experience and abstraction from actual usage events. Places are components of more complex symbolic structures, such as pointing and placing constructions.

The present article analyzes further dimensions of the Place symbolic structures, using data from Argentine Sign Language (LSA). Particularly, we focus on Places that create or track different kinds of reference from perceptually accessible entities in the ground to anaphoric referents in discourse. We suggest that deictic and anaphoric constructions, which incorporate Places, are aspects of the same conceptual system and that there is a continuum of Place symbolic structures in signed languages that varies in terms of subjectification. We also explore the way these different Places may function as reference points within larger constructions with the goal of providing mental access to related referents, which are the intended focus of attention.

In section “Cognitive Grammar,” we offer a brief background in the basic concepts of CG that are used in our analysis. Section “Pointing and Places” describes our account of pointing and of Places and introduces our proposal of the continuum of Places. In section “Places and Reference Points in Discourse,” we examine the use of Places and reference points in discourse. In section “Discussion,” we discuss Places in terms of subjectification, examine the implication of Places for infant pointing, and explore Place in relation to the development of demonstratives into grammatical markers. In the conclusion, we offer a summary of our findings and suggest areas deserving further research.

## Cognitive Grammar

We adopt CG as our theoretical framework for examining the conceptualization of space. The central claim of CG is that only three structures are posited (Langacker, 1987): semantic, phonological, and symbolic. *Semantic structures* are conceptualizations exploited for linguistic purposes. *Phonological structures* include sounds, gestures, and orthographic representations. *Symbolic structures* are the association of phonological and semantic structures such that one is able to evoke the other. The structuralist category of morpheme is viewed as a structure with zero symbolic complexity that has undergone progressive entrenchment and become established as a more or less conventional unit within a language community.

CG claims that lexicon, morphology, and syntax form a continuum of symbolic assemblies comprised of phonological structures, semantic structures, and the symbolic links between the two (Langacker, 1987). Symbolic assemblies vary along two dimensions: schematicity and complexity. *Schematicity* pertains to level of detail or precision. Schematic elements are elaborated or instantiated by more specific elements. Schematicity is, therefore, relational: An element is schematic to a more specific elaboration, and schemas are immanent in these more detailed instantiations. Schematic elements emerge as the result of the cognitive ability to extract and reinforce commonalities across multiple experiences. Symbolic structures also vary along a dimension of complexity. Symbolic structures combine with other symbolic structures to form complex symbolic assemblies. Constructions are symbolic assemblies, composed of component symbolic structures integrated to form a composite structure (Langacker, 2008).

In CG, conceptualization is seen as being “both physically grounded and pervasively imaginative” (Langacker, 2008, p. 539). Thus, grammar incorporates the full scope of our conceptual world and of the physical and spatial world within which we interact with other entities. CG adopts a conceptual semantics based on embodied cognition. Meaning is conceptualization that is grounded in our sensory and physical interactions with the world.

The experiential and embodied nature of cognition is reflected in conceptual archetypes and idealized cognitive models that feature prominently in the organization of grammar. *Conceptual archetypes* are experientially grounded concepts, such as a physical object, an object in a location, an object moving through space, seeing something, holding something, exerting force to effect a desired change, a face-to-face social encounter (Langacker, 2008). One conceptual archetype important to the analyses being offered here is “the common everyday occurrence of physically pointing to something” (Langacker, 2006, p. 34), which is arguably the baseline conception for nominal grounding (Langacker, 2016b). Another conceptual archetype consists in the organization of a scene into a global *setting* and mobile *participants*. “At a given instant, each participant is found at some location. A location is part of the setting (any point or area within it).” (Langacker, 2008, p. 355). This conceptual archetype is manifest in the *stage model*. The term evokes viewers watching action on a stage. We cannot observe the entire auditorium and its audience, the entire stage, and all the actors and action. Therefore, viewers must focus and direct their attention: From the maximal scope of their visual field, they attend only to certain elements, and within that more narrow scope, they focus on specific actors and their actions. This visual *perceptual* description is more than merely a metaphor. The embodied view of cognition claims that our *conceptual* organization also reflects a maximal and *immediate scope* of conception within which certain elements are profiled. A linguistic expression’s *profile* is the focus of attention within its immediate scope.

*Reference point* phenomena are ubiquitous in our experience of the world (Yamanashi, 2015). Reference points rely on our ability to direct attention to a perceptually salient entity as a point of reference to find some other entity, the target (Langacker, 1993). Each reference point provides access to a set of potential targets, called the reference point’s dominion. Reference points form the conceptual basis of many constructions, including possessives, topic constructions, metonymy, and pronominal anaphora. Reference point constructions have been shown to play a significant role in the grammars of signed languages (Wilcox and Occhino, 2016; Martínez and Wilcox, 2019).

The *ground* plays a pervasive and essential role in grammar. The ground consists of the speech or sign event, the participants, their interaction, their knowledge, and the time and place of the communicative usage event. The ground features in grammar through *grounding elements*: symbolic structures that specify the status of a nominal or a clause in relation to the ground. For nominals, the primary epistemic concern is identification (Langacker, 2017). Nominal grounding, such as demonstratives, articles, and certain quantifiers, directs the interlocutor’s attention to an intended discourse referent (Langacker, 2008; Martínez and Wilcox, 2019). Clausal grounding indicates whether a profiled occurrence has been realized by locating it in relation to the speaker’s or signer’s conception of reality, for example, by marking tense and modality (Langacker, 2017). Grounding is, thus, a deictic referential strategy: A deictic expression is one that includes some reference to the ground (Langacker, 1987, p. 126).

All of these principles and models are integrated in discourse. Discourse consists of *usage events*, specific instances of actual language use. Usage events consist of both poles of a symbolic structure: semantic (conceptualization) and phonological (expression). The conceptual pole of usage events “includes the expression’s full contextual understanding – not only what is said explicitly but also what is inferred, as well as everything evoked as the basis for its apprehension” (Langacker, 2008, p. 458). The expressive side consists of the full phonetic detail of an utterance. We do not limit usage events to only a single modality: A usage event includes *all perceptible detail*. Usage events have no particular size; depending on level of analysis, a usage event may be a word or sign, clause, conversational turn, or an extended discourse.

Discourse takes place in a discourse space comprising “everything intersubjectively accessible to the interlocutors as the basis for communicating at a given moment in the flow of discourse” (Langacker, 2016b, p. 108). Intersubjective accessibility here means both conceptual accessibility, that which is in the immediate scope of each interlocutor’s conceptual space, and perceptual accessibility. Perceptual accessibility includes the ground and the immediate physical context: that which is visibly accessible to the interlocutors.

For signed language discourse, the critical point to recognize is that space plays a role both conceptually and expressively. Signers point to and use physical locations in space to achieve intersubjective reference in discourse. All language users conceptualize space, and thus, space is meaningful in spoken and signed languages, but only signed languages incorporate physical space into their form. The significance of this dual role of space is revealed throughout this paper.

## Pointing and Places

In the sections that follow, we present a variety of discourse excerpts from LSA, which incorporate pointing. We analyze pointing as a construction (Wilcox and Occhino, 2016; Martínez and Wilcox, 2019). Pointing constructions consist of two component symbolic structures: a *pointing device* and a *Place*^3^. Both component structures of the pointing construction are symbolic structures consisting of a form, the phonological pole, and a meaning, the semantic pole. One type of pointing device is an index finger, but others may include hand(s), eye gaze, mouth or nose pointing, and body orientation. The pointing device functions to direct attention; this is its schematic meaning. The schematic semantic pole, thus, is dependent, making reference to some autonomous element that is the focus of attention. This is the function of the Place symbolic structure; its semantic pole is the thing referred to, and its phonological pole is the spatial location in the current ground of that referent.

**FIGURE 1:**
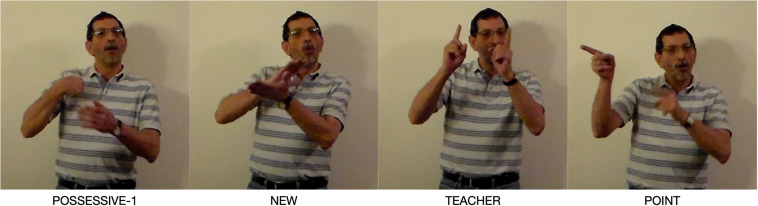
Proxy-antecedent construction.

Place structures play a role in a variety of grammatical constructions in LSA and other signed languages. These Place structures are typically quite schematic semantically and phonologically. They acquire full contextual meaning and a specific spatial location in the context of a usage event. One example [A] of the use of the Place symbolic structure is in proxy-antecedent constructions (Wilcox and Occhino, 2016). A proxy-antecedent construction from LSA is shown in Figure 1 (Martínez and Wilcox, 2019)^4^.

**FIGURE 2:**
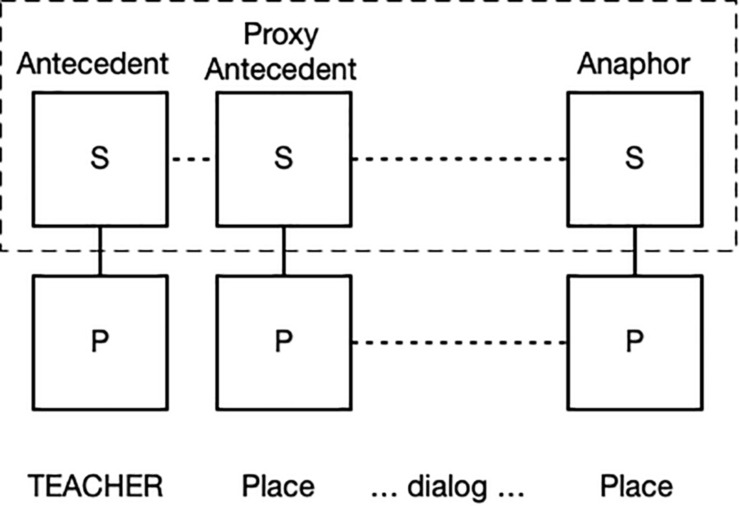
Proxy-antecedent construction.

The full nominal clause is formed by a possessive (POSS1), an adjective (NEW), a noun (TEACHER), a point to a location in the right of the signing space [POINT(right)], and a relative clause starting with SAME in which there are two more pointing signs. The ungrounded noun TEACHER provides the type specification. The first pointing sign occurs in a proxy-antecedent construction. The antecedent nominal (TEACHER) is grounded deictically by the possessive “my.” The proxy-antecedent construction associates the nominal antecedent with a Place, whose schematic meaning is elaborated by the nominal “my new teacher.” The proxy-antecedent construction also carries as part of its conceptual base the meaning that the antecedent will be used later in discourse. This occurs in the relative construction: two pointing signs are used to refer back to the antecedent “my new teacher.” Both use the same pointing device to direct attention to the Place on the right of the signer, referring anaphorically to the same antecedent. Figure 2 depicts this construction. A dotted correspondence line shows that the phonological pole (P) of the two Places structures share the same location in space. Correspondences lines also indicate that the antecedent (TEACHER), the proxy-antecedent (Place), and the anaphor (Place) refer to the same entity; they are coreferential. The dashed rectangle indicates overlap of the three in conceptual space.

### The Continuum of Places

The Places examined so far have been grammatical structures functioning to mark proxy-antecedent and anaphor relations. The phonological location of the proxy-antecedent and anaphor Place has nothing to do with actual entities in the spatial environment; the phonological location of Place is entirely specified by the grammar of LSA.

This is not always the case. Signers, like speakers, point to objects in the current discourse environment. It is important to understand the relationship between pointing to physical objects in the spatial environment and pointing to more abstract entities, such as proxy-antecedents and anaphors, whose location is determined by the grammar. Talmy (2018, p. 1) describes the distinction we wish to make as two domains of linguistic reference, “those traditionally termed anaphora and deixis.” He goes on to describe these domains: “Broadly, an anaphoric referent is an element of the current discourse, whereas a deictic referent is outside the discourse in the spatiotemporal surroundings. This is a distinction made between the lexical and the physical, one that has traditionally led to distinct theoretical treatments of the corresponding referents.” Talmy proposes that these two domains of reference engage the same conceptual system.

Engberg-Pedersen (1993) describes deictic and anaphoric frames of reference in signed languages. In deictic reference, the signer points to entities or locations in the context of the utterance. The frame of reference is dependent on the actual locations of those entities or locations. Consequently, if the signer or the entities change their location, the deictic frame of reference changes. Anaphoric frames of reference are independent of the utterance context and, thus, do not change.

Within our approach, we would say that these two domains of reference are not distinct categories; rather, they form a continuum. In order to understand the role of Place along this continuum, we must introduce the concept of “immanence.” Immanence has been a central concept in CG since its inception (Langacker, 1979). Immanent means “contained within” or “lies within.” Immanence plays a ubiquitous role in grammar, both semantically and phonologically. As we have seen, some units of language are schematic relative to others. Schematic meanings “are immanent in (i.e., they “lie within”) those of instantiating expressions, which elaborate them (“flesh them out”) in their own individual ways” (Langacker, 2009b, p. 14). The abstracted commonality of a type, such as “dog,” is immanent in the conception of any instance of dog. Immanence forms the basis for analyzing a host of expressions, including possessives, epistemic vs. root modality, and grammatical categories.

In all of these cases, the relationship is between the degree of attenuation of semantic units. The classical case is the relation between “going” to mean spatial movement and “go” marking future time. As described by Langacker (2008, p. 538), “In the former case, the conceptualizer scans through time by way of tracking the subject’s movement through space. On the future interpretation, this subjective temporal scanning occurs independently of any conception of spatial motion. It is merely a way of mentally accessing an event’s location in time.” This dynamic semantic relationship between more and less attenuated units is reflected in the concept of subjectification, a semantic shift in which an entity originally construed objectively comes to receive a more subjective construal, which we discuss in more detail in section 5.

We apply these concepts not only to the semantic pole of symbolic structures, but also to the phonological pole. In our usage-based approach, all linguistic units, including phonological units such as location, are abstracted by language users from actual usage events. The units abstracted are immanent in these usage events and motivate new expressions. We also assume that language is grounded in sensory and physical experience in which embodied cognition and experiential conceptual archetypes are fundamental. One conceptual archetype that is central to our proposal of Place is *an object in a spatial location*: this is, in fact, the archetypal source of Place. In pointing to physical entities in a usage event, the conceptualizer produces a pointing construction. Setting aside the pointing device for now, the entity, the thing referred to, is the semantic pole of the Place component of that construction, and the entity’s spatial location is its phonological pole. Of course, we point to or otherwise direct attention to any number, in fact an unlimited number, of entities in the environment. As a signer perceives and produces more of these usage events, she abstracts away from the specifics of any particular entity and its location, developing an ever-more schematic concept of directing attention to an *entity* in a *location*. This is the Place symbolic structure, in which the “entity referred to” is the schematic semantic pole and “some spatial location” is the schematic phonological pole.

Thus, Place symbolic structures are abstracted from actual usage events – in this case, the archetypal usage event being pointing to a physical object in a spatial location. Conceptualizers schematicize these usage events, arriving at a conception representing a higher level of abstraction. This higher level of conception is a schematic Place, which has neither a specific meaning nor a specific spatial location; rather, it associates a schematic meaning with a schematic phonological location. In use, the schematic meaning and the schematic location are instantiated, resulting in a fully contextualized Place symbolic structure.

Schematicity is not an all or none affair; it is a matter of degree, and the path involves attenuation. If pointing to a physical object in a location is the conceptual *baseline*, there are various ways in which this baseline can be *elaborated* (Langacker, 2016a, 2019). One elaboration involves the temporal stability of the object and its location. Suppose that you are sitting in a coffee shop with a friend who is drinking a cappuccino. She points to the cup and says, “This is the best cappuccino I have ever had.” She then leaves for a moment, and when she returns her cappuccino has disappeared (probably the waiter thought she was finished and took the cup away). She can point to the location of the now missing cup and say, “Where’s my cappuccino?” Now, she is pointing to the Place that was immanent in the cup’s spatial location. Even though the cup is no longer physically present in this location, your friend and you remember that it was. In another elaboration, one might return after many years to the house where she grew up and say, “My father’s desk was here, my sister’s here” and point to their former locations. Here, the elaboration is even more attenuated both because it involves a longer expanse of memory, and for the interlocutor, it requires imagination. Imagination can be used by both the speaker/signer and the interlocutor in further elaborations, such as pointing to purely hypothetical or virtual entities.

To summarize, Places are symbolic structures consisting of a phonological pole (a location in space) and a semantic pole (the most schematic meaning of Place is “thing”). Places fall along a continuum starting from a baseline of real objects in the spatiotemporal surroundings, the conceptual archetype of Place. Various cognitive processes operate to yield elaborations of this baseline situation. The entity with which a Place is associated may disappear, requiring memory. The entity may be present but not within the signer’s or addressee’s perceptual field, such as a Place associated with the spatial location of a distant house. Entities and the Places associated with them may be real but imagined, as in the teacher example, or they may be abstract, such as two theories located in signing space for purposes of comparison. All of these elaborations beyond the baseline of a real, physically present object require additional conceptual resources.

The entity with which a Place is associated may attenuate completely. All that is left is the Place (which was always immanently present). In this case, the meaning has become almost entirely schematic because the Place is not associated with any actual entity until it is used in an utterance. Its phonological location is largely schematic as well. Both the meaning and the location – the semantic pole and the phonological pole – of the Place are specified by the grammar of the language (although certainly contextual and pragmatic influences still may remain, e.g., focused referents may appear on the signer’s dominant side); the Place is fully instantiated semantically and phonologically in a usage event. Our claim is that this continuum captures both deictic and anaphor systems of reference, and that Place symbolic structures span the entire continuum.

Finally, we note that in our usage-based view, phonology is not a static list of *a priori* elements (in this case specified locations), but is instead dynamic, developmental, and emergent. As users visually track, point to, and direct conceptual attention to some physical entity, they build up a symbolic structure that becomes increasingly schematic the more they direct attention to different entities: The specific entity generalizes and attenuates to “thing,” and the location of the entity attenuates to “location.” That symbolic structure is a Place. The Place symbolic structure can now be recruited in more abstract uses, such as marking remembered or imagined entities, person reference, demonstratives, proxy-antecedents, and nominal components of directional verbs.

Last, our cappuccino-drinking friend reveals another significant aspect of Place structures. Although she directed attention to the Place of the cappuccino cup, the cup was not the ultimate target of attention. She had a motive for directing attention to the cup: in the first instance, to make a comment on its taste. Pointing to the Place established a topic, and her spoken utterance constituted a comment. This is a reference point construction. We see this function of Places in many of the following examples.

**FIGURE 3:**
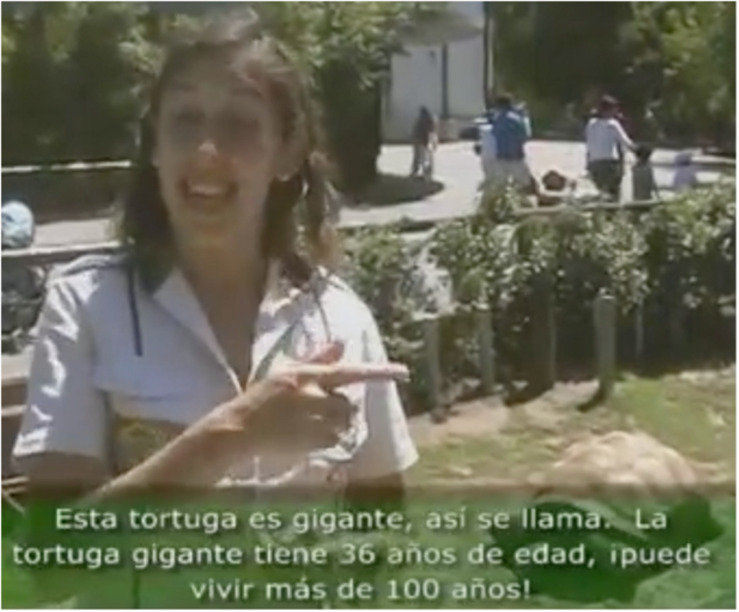
Place associated with physically present animate entity.

An example [B] of pointing to a Place associated with a physical object in the environment occurs in a video produced to introduce children to animals in the zoo. The signer, Eliana, is on location in the zoo explaining about the Tortuga Gigante “Giant Turtle.” Typically, a signer would select an area on the dominant signing side, in this case, the signer’s right side, to introduce the main topic of a discourse. Here, the signer is standing in front of the area in which the turtle is located, but it is on her left. She orients her body to the left, points to the left, and signs TURTLE GIANT TO-BE-CALLED and then again points to the left (Figure 3). Thus, the high perceptual accessibility of the actual spatial location of the turtle’s Place on the left of her signing space motivates her choice to establish the main topic of her discourse (the giant turtle) on her non-dominant side. This Place is maintained throughout her discourse.

**FIGURE 4:**
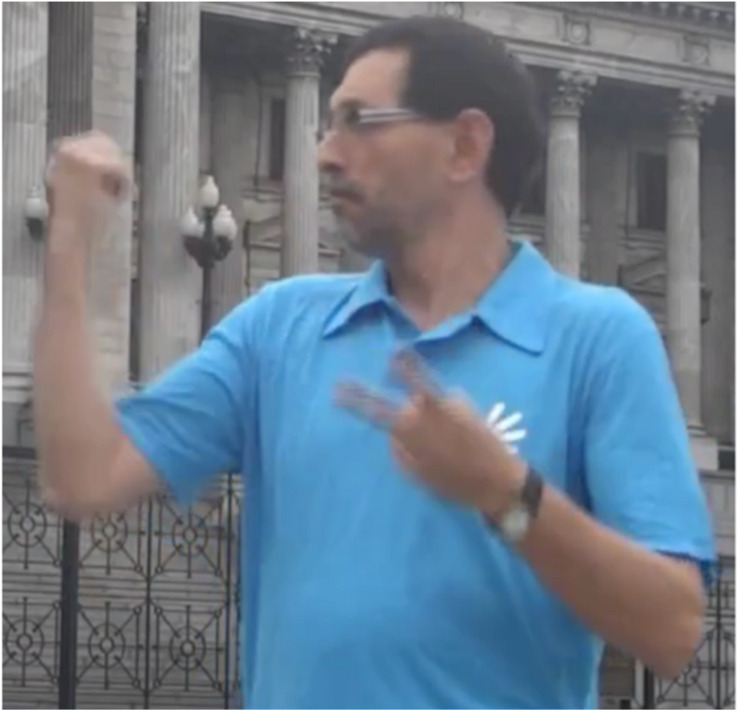
Place associated with physically present non-animate entity.

In another example [C], Pablo and Alejandro, members of the Movimiento Argentino de Sordos (MAS), have organized a demonstration to support a bill on the national recognition of Argentine Sign Language. They also prepared a video to describe strategies for explaining the linguistic problems of the deaf community in Argentina to hearing people unacquainted with the issues. Alejandro says that demonstrators should not talk to the press; instead, they should let the leaders communicate with the press, not because they don’t want demonstrators to express their ideas, but because they have strategies that will make an impact on the people. Then, he says that Pablo will give an example. In the fragment we analyze, Pablo introduces the problem (hearing people have a medical, not a cultural, view of deaf people). He then points (thumb-point) to the building of the National Congress, directing attention to it as a Place (Figure 4).

#### Places and Reference Points

When signers point to turtles or buildings to direct the interlocutor’s attention to an entity’s Place, they do so for the purpose of establishing mental contact with another entity. They are pointing to Places in order to create conceptual reference points.

In the giant turtle example, pointing to the turtle serves as a deictic strategy, along with the descriptive strategy of naming the entity, to produce a grounded nominal (Martínez and Wilcox, 2019). The signer then goes on to describe various characteristics of giant turtles: They can live more than 100 years, they have hard shells and scales on their legs, etc. The semantic pole of the Place, the turtle, serves as a reference point, and its dominion is the conceptual region to which it provides access: in this case, to the set of characteristics of giant turtles.

The discourse that continues in the Alejandro and Pablo example also reveals the use of Place to create reference point constructions. Pablo points to the building not for the purpose of directing attention to the building itself, but to establish a reference point, which he then uses to continue the discourse by talking about hearing legislators and their views of deaf people. Pablo directs attention to the building, establishing its Place as a reference point for the purpose of affording mental access to a target. In this case, Pablo ultimately intends to direct the interlocutor’s conceptual attention to the legislators, their views, and legislative activity that takes place in the building. Another way of describing this is that the legislators and their activity is the reference point target. The choice of spatial location (i.e., a phonological location) for establishing a new discourse referent in these examples is not randomly selected. The signers use perceptually accessible entities in the current physical environment (i.e., the ground) – the Places of the turtle and the building of the National Congress.

### Places and Placing

In addition to using Places as simple reference points, signers also incorporate Places as components in complex placing constructions. Continuing his narrative [C], Pablo explains that, because of their ideology, hearing people regard deaf people as mentally challenged, not equal to hearing people, mute, incapable.

To express this Pablo produces the sign PERSON, but rather than articulating it at an unmarked location in signing space, he signs it in the spatial location of Alejandro. We analyze such constructions as *placing* (Martínez and Wilcox, 2019). In placing, a sign is produced at a specific meaningful location in space. We identify two types of placing: create-placing, in which a new meaningful location, a Place, is created, and recruit-placing, in which the signer produces a sign in an existing Place. In this case, Pablo recruits a Place associated with Alejandro^5^.

**FIGURE 5:**
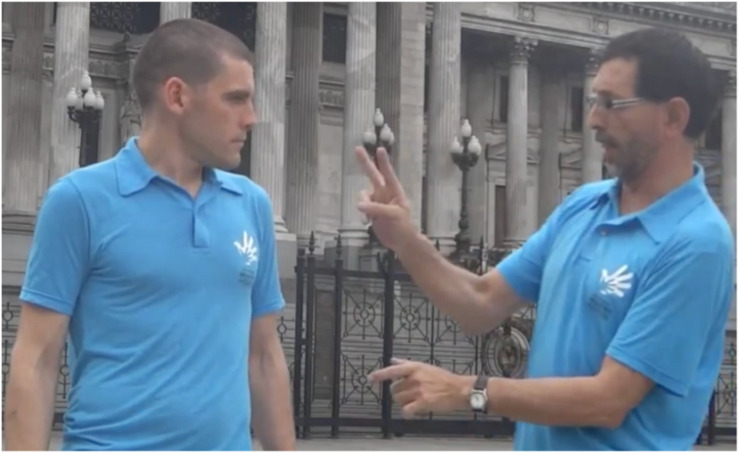
Placing construction as a component of a simultaneous construction.

This placing construction is a component in a larger, simultaneous construction (Figure 5). While Pablo continues to hold the placed sign PERSON with his non-dominant hand, he signs DEAF TO-SEE DEAF with his dominant hand. Because DEAF is a body anchored sign, unlike PERSON and TO-SEE, it cannot be placed. The verb TO-SEE is produced with a path movement moving from Pablo toward Alejandro. Pablo then lists the negative characteristics (mentally challenged, incapable, etc.).

For this entire discourse segment, Pablo is the conceptualizer. TO-SEE in the sense used here is not a perceptual verb; it is, rather, a verb of cognitive activity. TO-SEE means “to regard as” or “to think of,” and in this use, it expresses the cognitive activity of categorizing: hearing people categorize deaf people as those who are incapable, etc.

In the previous discourse segment, Pablo placed PERSON on his own body as a rhetorical device so as to frame hearing people, such as the legislators in the legislative building to which he has just pointed, as neutral addressees, lessening the tensions between hearing and deaf people – in effect saying “people in general” (Martínez and Wilcox, 2019). Because of the previous placing construction, Pablo is a type – hearing people. The conceptualizer of TO-SEE is “hearing people” – a virtual conceptualizer. Although TO-SEE is directed at Alejandro, he is not the object of categorization. In order to understand who is the object of categorization, we have to unpack two further constructions.

Looking at only the semantic poles, we see that, in the first construction, the semantic pole of the lexical sign PERSON is a type specification. When it is placed, it integrates with the semantic pole of Alejandro’s Place. However, the semantic pole of this Place is not Alejandro as an undifferentiated whole. Rather, Alejandro’s Place serves as a reference point, affording mental access to a dominion of targets, in this case of characteristics associated with Alejandro. We do not yet know which of those target characteristics are relevant. With Alejandro as a referent point, the targets could be Alejandro’s gender, his hair color, his clothing, or any number of other characteristics. Which is the selected target? In the discourse scene we are describing, the most salient target is Alejandro’s deafness. We can confirm this because it is also the characteristic explicitly mentioned when Pablo signs DEAF TO-SEE DEAF. The first construction, thus, integrates the component type specification PERSON with Alejandro’s Place, specifically the target “deaf” of the Place reference point, to create the composite construction “deaf people.”

**FIGURE 6:**
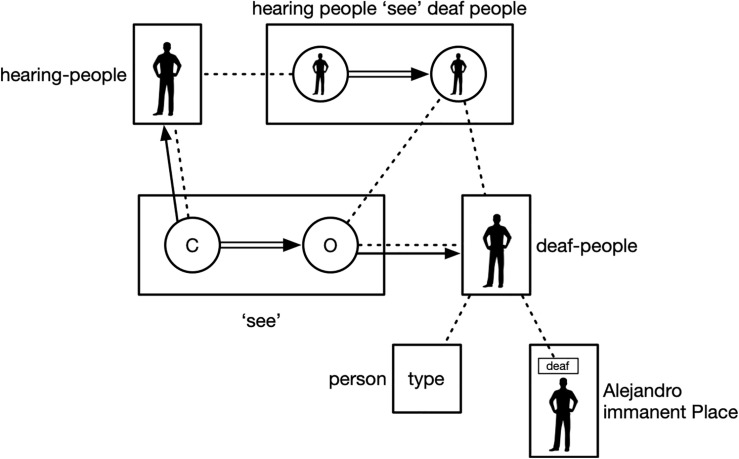
Complex simultaneous construction.

This composite construction is then a component in the higher-level construction that integrates “deaf people” with TO-SEE. TO-SEE is a cognitive activity verb with two schematic semantic elements: the categorizer and the object of categorization. These two elements are the semantic poles of two Place structures^6^. The first schematic Place, the categorizer, is elaborated in the prior discourse frame when Pablo uses the placing construction to present himself as a hearing person; it is hearing people who are doing the categorization. The second schematic Place, the object of categorization, is elaborated by the composite construction “deaf people,” producing the complex construction “hearing people see deaf people” as incapable, etc. Figure 6 depicts the semantic side of these constructions. Dotted lines indicate correspondence or conceptual overlap; filled lines with arrows indicate elaboration.

As a result, in this composite construction, we have virtual hearing people categorizing virtual deaf people – both of which are represented by real people (Pablo and Alejandro) in the discourse ground. The virtual deaf people are evoked by Alejandro’s Place when integrated with the type specification PERSON. The conceptualizer of TO-SEE is also a Place (Pablo’s), which has been semantically extended through the placing construction in the previous discourse frame to create virtual hearing people. Thus, Places associated with physical entities in the ground (Pablo as signer, Alejandro as one of the interlocutors) play essential roles in the component structures that go into forming this complex construction.

Looking at the phonological side of these constructions, we see comparable complexity. The relevant issue is the phonological poles of the various Place structures, which is their locations. The phonological location of PERSON is schematic, which is what permits it to be placed. When PERSON is placed, the phonological location of Alejandro’s Place elaborates its schematic phonological location. The two schematic elements of TO-SEE are Places. Pablo’s prior placing construction elaborates the phonological location of the first schematic Place, the categorizer, with the phonological location of his Place. The schematic phonological location of the second Place, the object of categorization, is elaborated by the phonological location of Alejandro’s Place.

Another way to view this complex construction is in terms of conceptual overlap. The semantic pole of Alejandro’s Place conceptually overlaps with the semantic pole of the placed sign PERSON as does the semantic pole of the object of TO-SEE: all three map to the same entity in conceptual space, deaf people. This *conceptual* mapping or overlap is achieved by *phonological* overlap: The phonological pole of all three structures are articulated at the same location in space in the discourse ground, the phonological locations of the Place structures.

Finally, this analysis reveals a complex level of grammatical iconicity grounded in conceptual archetypes. Participants (hearing people, deaf people) in an interactional setting are phonologically represented by the locations they occupy in Place symbolic structures. The subjective cognizing activity on the part of the categorizer (depicted by the double-line arrow in Figure 6) is phonologically represented as a path movement from the categorizer (hearing people) to the object of categorization (deaf people).

## Places and Reference Points in Discourse

We have shown that pointing constructions can incorporate Place referents in the physical environment. These deictic pointing constructions integrate with grammatical constructions and reference point constructions to create extended, cohesive discourse.

### The Life of Quinquela

**FIGURE 7:**
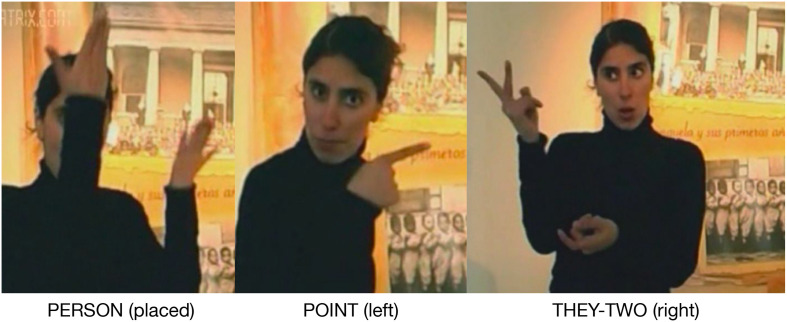
Placing and pointing constructions in discourse construction.

The next examples are taken from a video describing the life of Benito Quinquela Martín (1890–1977), an Argentine painter born in La Boca, Buenos Aires. The signer, Mercedes, is standing in front of a photo of the orphanage where Quinquela spent his early years. In this discourse excerpt [D], the photo of the orphanage, which is behind and on the left of the signer, is a recruitable Place. The signer uses a placing construction with PERSON in proximity of the photo and its Place and several pointing constructions using this Place as a component (Figure 7).

She then points to this Place, directing attention to it as a reference point. The target of the reference point, the reason why she points to the photo, is the situation of Quinquela’s life during this period, including the fact that his parents abandoned him at a young age. The signs expressing the target occur in this left Place. Placing a sign in the same Place as a previous reference point is the grammatical mechanism by which targets are identified and associated with their reference point (Martínez and Wilcox, 2019).

The signer continues her narrative, explaining that, at the age of six, Quinquela was adopted, still using the left Place for this phase of his life. She then introduces a new phase. For this new period, the signer reorients her body to the right and uses the right signing space for this portion of her narrative^7^.

She then signs OTHER, produced with an index finger and an arc movement toward the right. Although OTHER is a lexical sign, it also functions as a pointing device in a pointing construction, which creates and directs attention to a new Place with a phonological location on the signer’s right. She then places the sign THEY-TWO in this right Place. This Place is also recruited as a reference point to introduce a series of targets, aspects of Quinquela’s life with his adopted family. An initial pointing construction and all of the non-body-anchored signs used in this portion of the narrative are placed in the right Place: The phonological pole is the right signing space, and the semantic pole is this phase of Quinquela’s life.

The signer introduces two Places in this excerpt. The first, on her left, is recruited from the Place of the photo, and the second, on her right, is a new discourse Place created by pointing and placing constructions. These Places are two components in a sequential-events construction. This construction is based on timelines commonly observed in signed languages in which time is metaphorically represented as movements among locations in space (Engberg-Pedersen, 1993; Winston, 1995; Nilsson, 2016). In this case, the construction is used to describe a sequence of events comprising the two phases of Quinquela’s early life. Thus, in this example, a pointing construction that incorporates an entity in the physical environment via a point to its Place integrates with a conventionalized grammatical construction to create a coherent discourse structure.

### The Order of the Screw

Our last example [E] from the life of Quinquela comes from a portion of the narrative in which the signer describes Quinquela’s *Orden del Tornillo* (Order of the Screw). In 1948, Quinquela created this Order with a playful name for men and women (mostly artists) who, in the eye of common people, live in a state of madness. All the people who were to become members of the Order received a screw with a warning: “This screw will not make you sane. On the contrary, it will prevent you from losing this luminous madness of which you feel so proud.”

**FIGURE 8:**
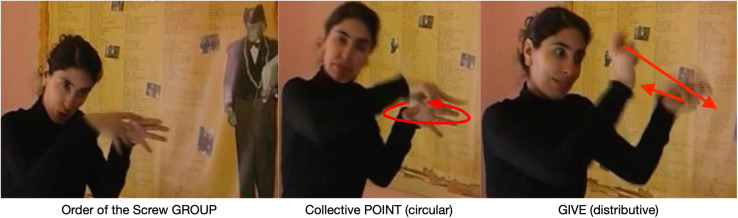
Placing and pointing constructions in simultaneous construction.

The setting has the signer standing near a poster describing the history of the Order of the Screw and showing Quinquela in his Order regalia, consisting of robes and a hat. The signer explains that Quinquela created this group and gave each member in the group a screw, which was the symbol indicating that they were now members of the Order of the Screw. She signs GROUP, a two-handed sign (Figure 8). While she holds her non-dominant hand in the GROUP sign, she then signs a circular point with her index finger. She then signs GIVE, a distributive verb indicating that Quinquela gave each member individually a screw.

The signer uses a simultaneous construction as we saw in the example of Pablo and Alejandro. The sign GROUP is produced near the poster, placing and recruiting the poster’s Place. The poster evokes the semantic frame of the Order of Screw, which serves as a reference point. The reference point could be used to evoke any number of targets, such as Quinquela, the regalia worn by Quinquela and the members, or the physical screw. By placing GROUP, the signer evokes one aspect of the Place’s semantic pole, the members of the Order construed collectively as a group. This collective construal is reinforced by the circular index pointing construction directing attention to this collective plural. In other words, GROUP directs attention to or profiles a collective entity; any substructure of that entity may be conceptually present in the immediate scope but unprofiled.

**FIGURE 9:**
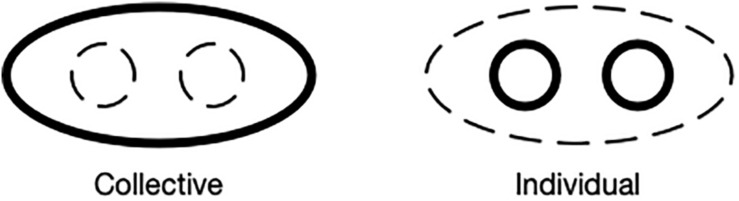
Collective vs. individual profile.

GROUP now serves as a new reference point, providing mental access to the giving event. By signing GIVE as a distributive verb, the signer changes the profile from the collective construal to one profiling the internal substructure of the group, its individual members. This profile shift has the effect of conceptual zooming from focusing attention on the collective to focusing attention on the individuals (Figure 9; Langacker, 1997).

As we saw for the sign TO-SEE in the Pablo and Alejandro example, GIVE is an agreement verb in which the final location of the verb’s path movement is a Place. The semantic pole of this Place conceptually overlaps with the individual members of the group^8^.

### San Martín and Argentine Independence

We have seen that Places associated with entities in the physical environment can be used as components in grammatical constructions. The methods of directing attention to Places include pointing, placing of signs, and body orientation. Often, the signer directs the interlocutor’s attention to a Place in order to use it as referent point, a conceptual stepping stone so to speak, affording access to a target, which is the intended focus of attention.

In this section, we examine Places that are created and used in discourse. We see that Places are created by grammatical constructions, such as pointing, placing, body orientation, and agreement verbs. Places serve as reference points for introducing and tracking referents in ongoing discourse. We also see that discourse Places are often “repurposed” by a series of reference point chains in which a reference point target serves as a new reference point in subsequent discourse.

**FIGURE 10:**
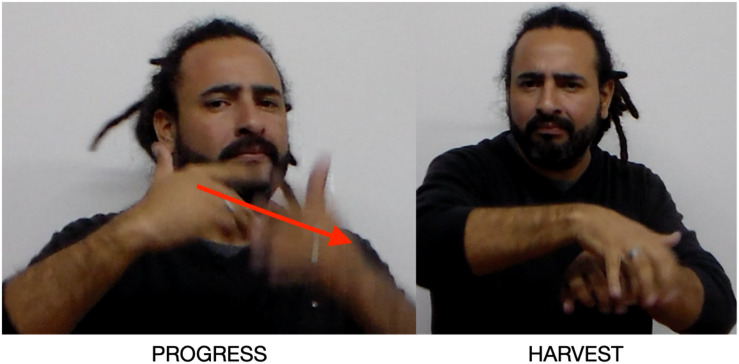
Argentina Place created by placing constructions.

The next examples come from a narrative signed by Diego Morales about the famous hero of the Argentine independence, José de San Martín (1778–1850). In the first [F], the narrator introduces Argentina with a proximal (downward) pointing construction (Martínez and Wilcox, 2019). He explains that San Martín lived in a small town called Yapeyú in the province of Corrientes, Argentina. San Martín had no opportunity to study and progress there, only to harvest the land or serve in the military. Two signs, PROGRESS (Figure 10) and HARVEST, are Placed in the left, creating an Argentina Place. In signing PROGRESS, the narrator also orients his body toward the Argentina Place.

**FIGURE 11:**
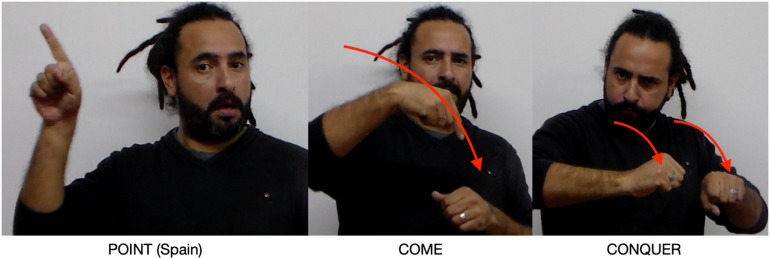
Spain Place created by recruiting the Spain Place.

He then comments that, a long time ago, Argentina was conquered by Spain. Spain is introduced with SPAIN, a body-anchored sign, and a pointing construction to the upper right signing space (Figure 11), creating a Spain Place: in this construction, the schematic semantic pole of this Place conceptually overlaps with Spain. He then signs the verb COME and CONQUER starting from the Spain Place and ending in the Argentina Place: again we see conceptual overlap with the semantic poles of the Spain place and the agent of COME and CONQUER and with the Argentina Place and the goal and patient, respectively, of these two agreement verbs.

We learn that San Martín had been living in Spain, where he became a successful military leader [G]. He returned to South America where he joined forces with another revolutionary, Manuel Belgrano. Two groups in Argentina had been battling, one opposed to the King of Spain, led by Belgrano, and another in support of the King. The narrator now “repurposes” the Spain Place and the Argentina Place. He does this by using the Spain Place as a reference point to refer to the King of Spain (the target); the Argentina Place is used to refer to two opposing groups in Argentina. Both of these reference point constructions express metonymic relations: the entity being referred to (the reference point Spain and Argentina Places) affords mental access to the intended target (King of Spain and two opposing groups).

**FIGURE 12:**
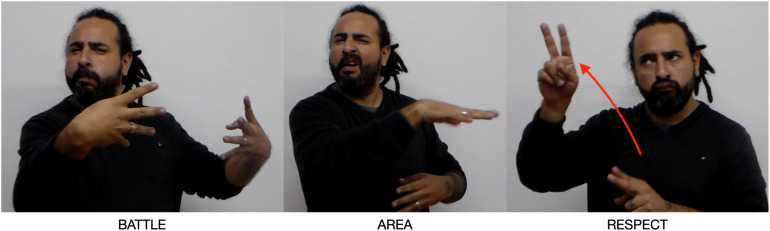
Argentina Place and Spain Place repurposed.

In addition to identifying the two opposing groups in Argentina as a unitary discourse element, the narrator creates two new subordinate Places, beginning with the signs BATTLE (Figure 12) and AREA: “In Argentina, two sides had been battling.” BATTLE is a two-handed sign. Each hand creates a new Place, dividing the previous left-hand Argentina Place into two new Places, one on the far left and the other center left. The narrative effect is to create two new discourse Places, both located in Argentina and each associated with a group engaged in battle.

San Martín discovers that one group, indicated by pointing to the left center Place, is the monarchists. The monarchists respect (give allegiance to) the King: RESPECT is signed toward the upper right Place formerly associated with the Spanish King.

**FIGURE 13:**
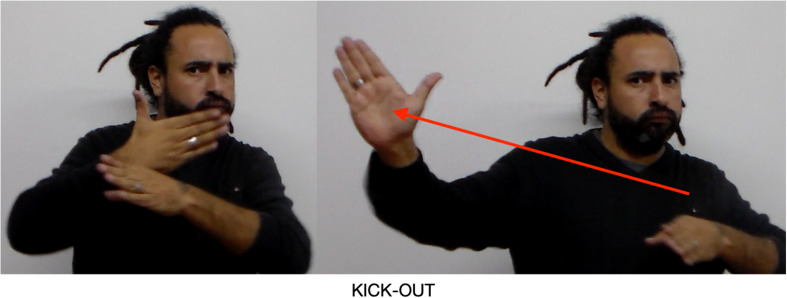
Agreement verb incorporating two Places.

The other is a group of revolutionaries opposed to the King (far left Place). They want to remove (KICK-OUT) the King. KICK-OUT is an agreement verb: the semantic agent, those who want to remove the King, is instantiated by the semantic pole of the revolutionaries Place (far left), and the semantic patient is the King, instantiated in the King Place (right and upper right). KICK-OUT moves from far left to upper right (Figure 13).

**FIGURE 14:**
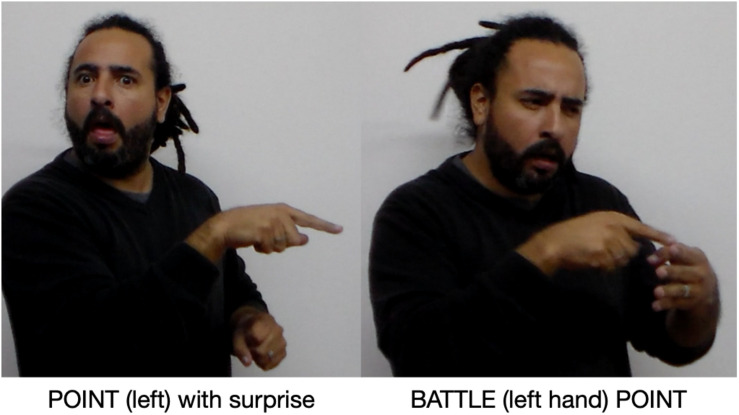
Reactivation of revolutionary Place.

The narrator then explains how San Martín came to meet Belgrano. First, he points to the left, reactivating the revolutionary Place, and indicates San Martín’s surprise at what he is about to learn with a facial display. He then signs BATTLE (Figure 14) again, but only with the left hand; simultaneously, his right index finger points to the hand signing BATTLE. It is among this group that San Martín finds Belgrano and realizes that they share revolutionary views. This discovery is expressed with a complex pointing and reference point construction. The right index point is the pointing device directing attention to the left hand, which has been recruit-placed to correspond to the left-hand Place: the left hand of BATTLE now conceptually overlaps with the revolutionary group. The narrator then uses this Place as a reference point, evoking as the target a new referent, Belgrano.

The reference point constructions in this small section of discourse are used creatively to repurpose the left Place with new referents, each conceptually connected with the previous one. As we saw in the Order of the Screw example, the effect is a zoom-in strategy by metonymic association:

Argentina > two battling sides in Argentina (revolutionaries vs. monarchists) > the revolutionaries > Belgrano.

## Discussion

As Engberg-Pedersen (1993) has so cogently observed, space is semantically loaded. We wish to point out that space is also phonologically loaded. Conceptual archetypes are the experiential basis of the semantic pole of linguistic items and constructions. For signed languages, conceptual archetypes are also the experiential basis of the phonological pole of linguistic items and constructions. In other words, the conceptualization of space is manifest semantically and phonologically in signed languages. This is especially important for Places because location in space is the phonological pole of Places.

In this section, we examine the semantic and phonological implications of subjectification on Places. Our analysis of Places has implications for the development of infant pointing. We also suggest that the various grammatical functions of Places mirror a pattern in the development of demonstratives described by Diessel (2006).

One aspect of the conceptualization of Places is the nature of their construal – from more objectively to more subjectively construed. These different construals form the basis of the continuum of Places described in section “The Continuum of Places.” We suggest that Places fall along a continuum of *subjectification*. As used in CG, subjectification concerns the asymmetry between objective and subjective construal. An expression or scene is objectively construed to the extent that it goes “on stage” as an explicit, focused object of conception. An element is subjectively construed to the extent that it remains “off stage” as an implicit, subject of conception. According to Langacker (2006, p. 21), subjectification can be thought of as a kind of semantic attenuation or “fading away”: in subjectification, a subjectively construed entity remains as a vestige of an objectively construed counterpart that was actually there all along, immanent in the latter.

An example of subjectification in adjective use is given in Athanasiadou (2006, p. 217):

(1) a. *The complete works of Shakespeare*.
    b. *He is a complete stranger to me*.

As Athanasiadou explains, in (1a), *complete* describes a spatial configuration rather than a property. It expresses an objective configuration. In (1b), the meaning shifts to a different type of quantification with a subjective construal. Here, the meaning of *complete* resides in the conceptualizing activity of the speaker. This conceptualizing activity is present in (1a), immanent in the conceptualization of the objectively construed spatial scene, but in (1b) it is used in abstraction from any objective spatial configuration (Athanasiadou, 2006).

In these examples, objective vs. subjective construal has pertained only to the semantic pole. Because signed languages are produced in visible space, the phonological structure of signed languages affords a second “layer” of construal and subjectification. It is this second layer that is central to the different construal of Places. In section “The Continuum of Places,” we identified attenuation as a factor that underlies the continuum of Places. One dimension of attenuation is the degree to which elements are objectively or subjectively construed with elaborate conceptual content inviting more objective construal.

Consider the following example (from Langacker, 2008), in which the arrow indicates a pointing construction:

(2) I want this [→] one

As Langacker (2008, p. 468) observes: “In addition to its signaling role, this gesture is part of the situation being described. The sentence describes a relationship in which the speaker goes onstage as a focused participant. Part of this onstage situation is the very fact that the speaker is pointing at something, and the object is specifically identified as what the speaker is pointing at.” This is the baseline Place associated with a physical object in the environment. Because of the elaborate conceptual content of the actual physical object, this Place is objectively construed.

An example in our data of more objective construal occurs when Pablo points to the legislative building. He directs mental attention but also (potentially) visual attention to the physical building in the current spatial environment – to the Place of the building. While he uses that Place as a reference point to direct mental scanning along a path leading to the target, the physical presence of the building invites an objective construal.

The functional difference between pointing and placing also figures in objective vs. subjective construal. Pointing directs attention, both perceptual and conceptual. When the signer points to the photograph of Quinquela in the Order of the Screw example, she directs attention to the photograph and its objectively construed Place. Placing, however, attracts attention rather than directing it (Martínez and Wilcox, 2019). This lowered focus of attention to the physical scene also lowers the objective construal. When she signs GROUP, the physical location of the photograph is no longer salient; the placing construction, which serves as a reference point, leaves behind only the mental operation of scanning to locate the intended target.

Places such as those used by Diego in the San Martín narrative show further subjectification. When Diego uses an upward location for Spain and a download location for Argentina, he puts onstage elements of the ground and general knowledge about the world (he is signing in Argentina, and recruits knowledge about maps and where the countries are), thus retaining a vestige of objective construal. The use of Places with directional verbs, for example, in the Pablo TO-SEE construction (“hearing people see deaf people as incapable”) and the distributive “giving” construction in the Order of the Screw example are more highly subjectified Places, used for the mental operation of locating referents in conceptual space. Finally, when Places are used in constructions such as the proxy-antecedent, as in the example of Pablo describing his new teacher, the physical location of the Place has no significance in terms of the physical environment other than to allow the signer focus the interlocutor’s mental attention (through the use of the proxy-antecedent Place) on the antecedent referent.

We have now arrived at the conceptual doorstep of creating and using Places in grammatical constructions: point to a location in space and map that location conceptually to some discourse element. Point or otherwise direct attention to that location in space (the Place) later in discourse and it becomes a component in a grammatical construction: anaphor, third person pronoun, agreement marker. In other words, when different instances of Places are used, an abstracted schema, a Place, emerges.

When a signer uses a pointing or placing construction to create a Place and then points to or uses that Place in subsequent discourse, the interlocutor is not instructed to direct visual attention to the Place, and in fact, nothing is visible in the Place. The interlocutor is only instructed to direct mental attention to the semantic structure of the Place. This then is one manifestation of the “complete disappearance of an objectively construed entity with retention of mental operations immanent in its conception” (Langacker, 2006, p. 29).

The same cognitive processes of subjectification and reference points are also implicated in the development of infant pointing. The pioneering work on deictic pointing in infants proposes two functions: protodeclarative and protoimperative (Bates et al., 1975). Summarizing and expanding on this research, Tomasello et al. (2007) observe that, in protoimperatives, infants point to objects they want or to request an action involving that object. Protodeclaratives not only are used for directing attention to something, but also for many different reasons, including remembering non-present events. In all cases, the infant invites the recipient to attend to the referent for a reason. To understand why the infant is pointing, the adult must understand both *what* the infant is directing attention to and *why* the infant is directing attention to it (the motive) (Tomasello et al., 2007)^9^. We claim that, in these pointing constructions, the *what* is a Place serving as a reference point, and the *why*, the motive, is one of the many potential targets in the reference point’s dominion.

Reference points serve as the basis for the analysis of topic constructions (Langacker, 2008). Infant pointing exhibits the same structure: “Pointing serves to establish a new topic, about which further things may then be communicated” (Tomasello et al., 2007, p. 719). In fact, we would claim that, in these cases, the reference point structure of pointing manifests a related type of conceptual archetype, searching and finding (Langacker, 2006). In order to understand a pointing construction, the interlocutor must search and find the motive for the point. In spatial searching and finding, a *search domain* is the spatial region in which the searched for entity is located. Prepositions, for example, reflect the search and find conceptual archetype. In locative expressions, such as *under the table*, the search domain is the spatial region to which a locative expression confines its subject.

Our claim is that, in pointing constructions, the search domain is the reference point’s dominion, the region in which the interlocutor must search for the motive for directing attention to the reference point. The person pointing is trying to do something. Why is the infant directing the adult’s attention to a Place associated with some object? In signed language Place constructions, why is the signer directing attention to an anaphor Place? In some instances, the answer is provided in accompanying language. In others, the interlocutor must discover the answer: the infant wants the object, or the signer is directing the addressee to search for the antecedent (by way of the proxy-antecedent).

Finally, we note that the function of Places mirrors the pattern described by Diessel (2006) in the development of demonstratives. Diessel observed that, in exophoric use, demonstratives focus the interlocutor’s attention on concrete entities in the physical world. When used in discourse, demonstratives focus attention on linguistic elements in the surrounding discourse context. These represent the two ends of our Place continuum. Further, Diessel notes that the communicative function of demonstratives extends from the physical world to discourse: “Demonstratives are not only used with reference to concrete entities in the surrounding situation, they may also refer to linguistic elements in the ongoing discourse” (Diessel, 2006, p. 481). Diessel claims that in both cases, the same psychological mechanisms are at work. We see this as the same conceptual underpinnings that unite the continuum of Places.

Diessel suggests a developmental path of demonstratives into grammatical markers:

(3) deictic DEM > anaphoric DEM > 3.PRO > pronominal clitic > agreement marker > Ø

We note that the functions Diessel has documented are much the same as those we have described for Places. Symbolic Place structures function as demonstratives (both exophoric and discourse), as anaphoric pronouns, as non-first person (but also first person) pronouns, and as agreement markers.

## Conclusion

We have examined the conceptualization of space in signed language discourse within the theory of CG. Symbolic structures are basic explanatory concepts in CG; lexicon and grammar form a gradation consisting solely in assemblies of symbolic structures varying in degree of complexity and schematicity. We have proposed that Places are basic elements of signed language structure, defining Place as a symbolic structure that associates a semantic pole (“thing”) and a phonological pole (location). Places acquire full contextual meaning and a specific spatial location in a usage event.

We suggest that our account of Places reveals new aspects of how space is semantically and phonologically conceptualized. Places provide a unified and natural account of signed language data that is often compartmentalized into separate cognitive systems. As we have seen, some sign linguists argue that pointing to or incorporating locations in the physical environment lies outside of language altogether and must be treated as part of a gesture system. We see no need to segregate the conceptualization of locations in space into distinct cognitive domains. Our primary claim is that Places unify deixis and anaphor. Rather than representing two distinct domains of reference, we suggest that they are ends of a symbolic continuum that varies in terms of subjectification.

The various functions of Places are accounted for with nothing more than core concepts of CG such as conceptual archetypes, schematicity, subjectivity, reference point constructions, conceptual overlap, and conceptual elaboration. We have, however, extended the use of these core CG concepts beyond the semantic pole of symbolic structures to the analysis of the phonological pole. For example, we have claimed that an object in a location is the conceptual archetype for Place. We would also suggest that conceptual archetypes are the experiential basis of basic phonological categories: a physical object (hand shape), an object in a location (location), and an object moving through space (movement). Further elaboration of these CG concepts could prove fruitful for the development of a cognitive phonology of signed languages.

CG has shown that reference point phenomena are manifest across a broad range of grammatical and discourse functions, including possessives, topic-comment, metonymy, and pronoun-antecedent relationships. We have shown that Places serve as both perceptual and conceptual reference points with many of the same grammatical functions.

Our analysis makes certain predictions for future research. The conceptual archetype for Places is a physical object in a location. This is also the archetype for noun: a physical object composed of material substance residing primarily in space (Langacker, 1987, 2008). Thus, the schematic meaning of Place is compatible with the schematic meaning of noun. This suggests that Places play a role in linguistic expressions of signed languages that incorporate the schematic conception of thing, such as nominals and verbal constructions. Concerning the latter, further research should be carried out to better understand the role of Places within so-called directional or agreement verbs, which incorporate nominal referents to the verb. CG treats agreement as multiple symbolization, a special case of conceptual overlap characteristic of all grammatical constructions (Langacker, 2009a). Place symbolic structures are, we suggest, the site of conceptual overlap in these directional verb constructions.

We have suggested that the different functions of Places result from increased and subjectification of the phonological and the semantic poles. One way in which subjectification is manifest is the diachronic process of grammaticalization. As we have shown, the patterns of semantic function of Place show notable similarities with the function and grammaticalization pattern report by Diessel (2006) for demonstratives. Although long-term patterns of diachronic change are difficult to study in unwritten languages such as signed languages, these patterns of change are attested at much shorter time-scale such as the verbalization of experience in narratives (Croft, 2010), suggesting a possible method for confirming these patterns.

Finally, we have suggested that conceptual elaborations, such as those that account for the semantic change from more deictic to more anaphoric reference, require increasing conceptual resources such as memory and imagination. This suggests that these more elaborated meanings are acquired later in development.

## Data Availability Statement

All datasets generated for this study are included in the article/Supplementary Material.

## Ethics Statement

Written informed consent was obtained from the individual(s) for the publication of any potentially identifiable images or data included in this article.

## Author Contributions

RM selected, transcribed, organized the data in LSA, and wrote the Supplementary Appendix. SW wrote the first drafts of each section of the manuscript, designed, and edited all the figures. Both authors contributed to the conception and design of the study, thoroughly analyzed, and discussed the data.

## Conflict of Interest

The authors declare that the research was conducted in the absence of any commercial or financial relationships that could be construed as a potential conflict of interest.

## Footnotes

1 We should point out that not all linguists working within the formalist tradition agree with this claim. Two prominent opponents are Quer (2011) and Wilbur (2013).

2 Blending is a process that operates over two mental spaces as inputs. Structures from each of the two input spaces is projected to a third space, referred to as the blend. The blend inherits partial features from each of the input spaces and also includes features that belong only to the blend.

3 We capitalize Place to indicate that the term applies to the entire symbolic structure. We use “location” as the term for the phonological pole of Place.

4 Information about data sources and methodology of coding is provided in Supplementary Appendix 1. Glossing conventions are listed in Supplementary Appendix 2. Full glosses of all examples are given in Supplementary Appendix 3. Citations to data glosses are given in brackets in the main text, e.g., [B] refers to data gloss B in Supplementary Appendix 3.

5 Martínez and Wilcox (2019) analyzed this as a create-placing construction. Because Pablo uses a Place, our analysis is that it is recruit-placing.

6 In a fuller treatment. we would analyze TO-SEE as an agreement verb. There is widespread disagreement among sign linguists about agreement. We adopt a cognitive-functional analysis of agreement (Barlow, 1999; Croft, 2013). Specifically, we treat agreement as multiple symbolization, a special case of conceptual overlap characteristic of all grammatical constructions (Langacker, 2009a). In signed languages, the conceptual overlap marking agreement is achieved via Places.

7 Winston (1993) suggests that orienting the body is a type of pointing; this is compatible with our analysis, in which the body is a pointing device used to direct attention to a Place (Wilcox and Occhino, 2016).

8 In this construction we see what would be, according to a formal approach, an agreement discord: GROUP is singular but GIVE is distributive, expressing the event of giving screws to multiple individuals within the group and not to the group. Following Barlow’s (1999) discourse-based treatment of agreement, we would say such “feature mismatches” suggest that agreement is providing information about the nature of the discourse referents rather than information about the morphosyntax of the controller. Thus, this example could be explained in terms of an association of two compatible properties (“collective” and “composed of individuals”) between the singular noun GROUP (the primary discourse referent) and the inflection of the verb GIVE (the secondary discourse referent). A fuller treatment of how a cognitive-discourse approach to agreement integrates with our Place analysis is beyond the scope of this paper.

9 Tomasello et al. make the important observation, with which we agree, that we cannot assume infants comprehend and produce their early pointing gestures with full adult-like meaning. They suggest we can assume, however, that infants may operate with some kind of “primordial, undifferentiated communicative intention that contains the basic structure, but not all of the adult details” (Tomasello et al., 2007, p. 715).
