# Supplementary material for: The Conceptualization of Space: Places in Signed Language Discourse
Source: Front Psychol. 2020 Jul 8;11:1406. doi: 10.3389/fpsyg.2020.01406 (PMC7360800; doi:10.3389/fpsyg.2020.01406)
Supplement: Supplementary file 1 [file Data_Sheet_1.docx]

**Appendix 1. Data and methodology**

Data consists of 5 videos produced by deaf LSA signers that belong to different genres: comment (Video 1), educational material (Video 2), political discourse (Video 3), and biography (Videos 4 and 5). The total length of the data is 37 minutes. Videos 1 and 5 belong to one of the co-author’s data (Martínez) collected as part of her ethnographic work within different spaces of the Argentine deaf community, while Videos 2, 3 and 4 are publicly available in DVD format and/or online. For the former, there are no physically present elements in the ground for the signers to incorporate to their discourse. On the contrary, the later were filmed in locations where there are physically present elements in the ground that could be incorporated into the signer’s discourse.

The data was completely transcribed using glosses (See glossing conventions in Appendix 2). We analyzed qualitatively the use of Place throughout each discourse. Then, we selected examples of different observed phenomena to be analyzed and discussed in the present article (See Appendix 3).

The following table gives basic information on each video:

| **Video N#** | **Topic of the video; genre; name of the signer(s)** | **Methodology** | **Available online** | **Data source for examples** |
| --- | --- | --- | --- | --- |
| 1 | My new teacher; comment; Pablo Lemmo | Topic: Talk about something that happened to you recently. Video recorded as part of one of the author’s ethnographic work within the Argentine deaf community. | No | A |
| 2 | The giant turtle; educational material; Eliana González | Non-elicited material: It belongs to a series of videos in LSA about animals in the zoo for kids. The signer was filmed in different locations inside the former Buenos Aires Zoo (nowadays called Ecoparque). Extracted from a DVD called “Learning at the Zoo”, by an NGO called CEA (Centro de Estudios y Acción para la Comunidad Sorda). | Yes <https://youtu.be/c1xUB2ZDdps> | B |
| 3 | Strategies for explaining the linguistic problems of the deaf community in Argentina to people not acquainted; deaf political discourse; Pablo Lemmo and Alejandro Makotrinsky | Non-elicited material: This discourse has been uploaded two days before a demonstration to support the bill on the national recognition of Argentine Sign Language. Pablo and Alejandro are two leaders of the Movimiento Argentino de Sordos (MAS). The video was filmed in front of the National Congress in Argentina. | Yes  <https://www.youtube.com/watch?v=cLoI4aKXbDg> | C |
| 4 | Life of Benito Quinquela Martín (1890 – 1977); Biography; Mercedes Pandullo | Non-elicited material: It belongs to a series of videos in LSA about the Argentinian painter Benito Quinquela Martín. The signer was filmed in different locations inside and outside the Benito Quinquela Martín Museum, which is placed in the neighborhood of La Boca (Buenos Aires, Argentina). Extracted from a DVD called “Learning at the museum”, by an NGO called CEA (Centro de Estudios y Acción para la Comunidad Sorda). | Yes  <https://youtu.be/-3WKoq-2nhk> | D and E |
| 5 | Life of José de San Martín (1778 – 1850); Biography; Diego Morales | Topic: Choose an historic or fictional character you like, and tell me everything you know about him or her. Video recorded as part of one of the author’s ethnographic work within the Argentine deaf community. | No | F and G |

**Appendix 2. Conventions for glossing**

| **Gloss** | **Meaning** |
| --- | --- |
| GLOSS | Closest meaning in written language to the meaning of the sign. E.g. TABLE. |
| GLOSS-GLOSS | A sign that needs more than one written word to give complete information on its meanings. E.g. LONG-TABLE. |
| G-L-O-S-S | Fingerspelling |
| [GLOSS GLOSS]-BODYright | The bracketed glosses indicate the extension of meaningful non-manual information. E.g. [HOW-ARE-YOU]-q (“how are you?”), [PERSON BORN HERE]-BODYright (“the person was born here”). The bracketed glosses in the first example indicates the signer uses non-manual information that corresponds to a question. In the second example, the construction is made with the signer’s body oriented to his/her right. |
| 1GLOSS2  1GLOSS  GLOSS1 | A sign that has information on person within its structure. E.g. 1TELL2 (“I tell you”); PRO1 (“I”). |
| POINT(location) | A pointing construction that uses a pointing device and is directed to a specific location within signing space. E.g. POINT(left) means that there is a pointing construction directed to a location in the left of the signing space. |
| GLOSS(location) | A sign that is placed in a specific location within signing space. E.g. TREE(right) means that the sign TREE is placed in a location in the right of the signing space. |
| GLOSS(inflection) | Change in the sign that adds grammatical information. E.g. perfective aspect (perf.), plural (pl.). |
| GLOSS+ | Repetition of a sign. |
| SAME(rel.) | Function word that introduces a relative clause. |
| ‹GLOSS GLOSS› | Embedded relative clause. |
| GLOSS(2H) | A sign that is made with two hands that usually is done with one hand, or a sign that -having both options- is done in the two-handed version. |
| PRO | Personal pronoun |
| POSS | Possessive |
| / | Pause |
| DH | Dominant hand |
| NDH | Non-dominant hand |
| HC | Hand configuration |
| *[information]* | Audiovisual information that is perceptually accessible in the ground and that is relevant for the discourse. E.g. *[There is an image of the painter behind the signer]* |

**Appendix 3. Selection of fragments extracted from the data**

| **A) My new teacher fragment (Video 1) / Timecode: 00:00 – 00:08** |
| --- |
| POSS1 NEW TEACHER POINT(right) <SAME(rel.) PRO1 1TELL2(perf.) POINT(right) TO-RESEMBLE POSS1 MOTHER TO-RESEMBLE POINT(right) > / YESTERDAY TO-BE-ABSENT(perf.) |
| *My new teacher, the one I told you resembles my mother, was absent yesterday*. |

| **B) The giant turtle fragment (Video 2) / Timecode: 00:15 – 00:29** |
| --- |
| *[There is a turtle behind and left to the signer]*  POINT(left) TURTLE [GIANT(left) TO-BE-NAMED(left) POINT(left) GIANT(left)]-BODYleft /  [AGE 36 YEARS-OLD]-BODYleft /  [CAN LIVE MORE 100 YEARS-OLD / VERY-LONG LONG-TIME CAN LIVE]-BODYright  [WOW]-BODYleft // |
| *This animal is called the giant turtle. It is 36 years old. It can live more than 100 years old, which is a very long time. Wow!* |

| **C) The ideology fragment (Video 3) / Timecode: 00:57 – 2:03** |
| --- |
| *[The building of the National Congress in Argentina is behind the signers. There are two signers in front of the camera. In this fragment, Pablo signs and Alejandro is next to him, to his right]*  Pablo: EXAMPLE CLEAR WHAT/ PROBLEM WHAT/ ARGENTINA SOCIETY ALL PERSON(pl.) HEARING / SECOND [POINT(back, to the building)]-BODY rotates right and behind, looks toward the building, and then rotates back to face the camera GOVERNMENT LEGISLATOR LEGISLATOR [DIFFERENT]-BODY rotates right and behind, looks toward the building, and then rotates back to face the camera GOVERNMENT PRO3pl THERE-IS WHAT/ PERSON(toward his own body) WRONG PERSON(toward his own body) NO(HC.5) / IDEOLOGY(up.right) POINT(downward, from above)+ IDEOLOGY(up.right) POINT(downward, from above)+++ / PRO3pl PERSON(pl.) HEARING AUTOMATICALLY TO-MEAN TO-SEE(center)  [NDH: PERSON(toward Alejandro)(hold)  DH: DEAF TO-SEE(toward Alejandro) DEAF]-BODY rotates right, signer looks where Alejandro is  IDEOLOGY(up.right) AUTOMATICALLY(up) TO-MEAN  NDH: FIRST  DH: MENTALLY-CHALLENGED POINT(to sign FIRST)  DISABLED / TO-BE-LESS TO-EQUAL TO-BE-LESS / TO-EQUAL DEAF-MUTE /  OR IN-A-WAY IDEOLOGY(up.right) POINT(downward, from above)++  TO-MEAN DEAF CAN’T TO-MEAN / [RIGHT]-q  IDEOLOGY(up.right) PT(downward, from above)+ PRO1 TO-LOOK-FOR WHAT/  IDEOLOGY(up.right) TO-CHANGE(up.right) POINT(downward, from above)  PERSON(towards his own body) TO-CHANGE(towards his own body) NO(HC.5)  IDEOLOGY(up.right) TO-CHANGE(up.right) / POINT(upwards.right)++ / POINT(upwards.right)+ TO-CHANGE(up.right) HOW/ MUST GOOD TO-GIVE-INFORMATION GOOD / |
| *The problem in Argentina is that all hearing people and the government have the wrong ideology about deaf people. The problem is not hearing people themselves, but what they think about us. They see a deaf person and automatically think that he/she is mentally challenged, disabled, deaf-mute, or less of a person. To their way of thinking, to be deaf means that the person is unable to do things, right? Well, it is their ideology that we want to change, not the people. And the only way to do that is by providing good information.* |

| **D) The orphanage fragment (Video 4) / Timecode: 0:56 - 1:57** |
| --- |
| *[Behind the signer there is a poster with two pictures of the orphanage where Quinquela spent his childhood]*  NOW WHAT(2H) / *[The signer moves one step to the right and her body orients to the left]*  [HISTORY MAN PERSON(left) POINT(left) / NOW LIFE 1TELL(left) PERSON(left) POINT(left)]-BODYleft. [CALLED(center) B-E-N-I-T-O LAST-NAME Q-U-I-N-Q-U-E-L-A M-A-R-T-Í-N / POINT(left) BORN BUENOS-AIRES(left) HERE(2H)+ 1890 POINT(left)/]-BODYcenter [POINT(left) PAINT PLACE FOR-A-LONG-TIME BOCA PLACE /]-BODYleft [POINT(left) PAST BORN FATHER MOTHER BORN DECIDE THEY-TWO ABANDON(perf.left) POINT(left) IN-THE-PAST/]-BODYleft.  [POINT(left) GROW-UP(left) STAY(left) PLACE(left) CHILD(pl) ABANDON(pl.perf.) PLACE(left) SAME *[signer moves one step closer to the poster]* SHOW POINT(circular movement toward picture of children in front of the building) //]-BODYleft. [GROW-UP(near the picture.left) UNTIL(left) SIX YEARS-OLD POINT(left)]-BODYleft.  *[Signer moves one step to her right]* OTHER(from left to right) MARRIED-COUPLE(right)  MAN WOMAN THESE-TWO(right) ADOPT(perf.left) POINT(right) /  *[Signer moves one step to her left]* THESE-TWO(right) FAMILY(right) POINT(right) WORK(right)  THINGS(right) WHAT-KIND-OF / BLACK ROUND-SMALL-OBJECT FIRE  GROUP-OF-OBJECTS MAKE-BAGS CARRY-BAGS-IN-SHOULDER(habitual)  POINT(right) /  *[Signer moves one step to her right]* POINT(left) SIX YEARS-OLD THEN SEVEN YEARS-OLD GET-INTO(left) SCHOOL(left) GET-INTO(left) CONTINUE(left) ABANDON(perf.left) FINISH(left) BECAUSE HAVE-TO(right.fast) GO(from left to right) WORK(right) TOGETHER(right) FAMILY(right) POINT(right) / WORK(habitual.right) THEN GROW-UP TEENAGER POINT(right) GO-TO(left) SHIP SHIPS-ARRIVE(left.2H.alt) WORK(left) POINT(left.circular movement) FOR-A-LONG-TIME(left) STAY(left) SAME TIME GO-TO(left and right.alt) GO-TO(right) DRAW(right) STUDY(right) DRAW(right). |
| *Now I’m going to tell the life story of this man called Benito Quinquela Martín. He was born in Buenos Aires in 1890. The constant topic of his paintings was the neighborhood of La Boca. When he was born, his biological parents abandoned him.* *He grew up in an orphanage, which is what we see in this picture. He was six years old when a married couple adopted him. This family worked with coal, making bags of this product and transporting it.* *He turned seven years old and got into school. He continued school until he had to drop out because he had to work with his family. As a teenager, we worked in the harbor for a long time and, in the meantime, he also studied drawing.* |

| **E) The Order of the Screw fragment (Video 4) / Timecode: 3:48 – 4:00** |
| --- |
| GROUP(left) POINT(left) CREATE(left) GROUP(left) CALLED(left) O-R-D-E-R  SCREW POINT(left) [GROUP(left hand)/POINT(circular mov. right hand)](left) POINT(left) SHOW[not fully completed sign] GIVE(multiple locations in the left) PRO1 SCREW GIVE(multiple locations in the left) EACH-ONE(multiple locations in the left) GIVE(multiple locations in the left)  GROUP(group) POINT(left) LIKE SYMBOL POINT(left) |
| *He created a group called The Order of the Screw, and gave to each member a screw, which was their symbol.* |

| **F) Early life of José de San Martín (Video 5) / Timecode: 00:00 – 01:01** |
| --- |
| NOW PRO1 1TELL2 PERSON(right) VERY-FAMOUS WOW RENOWNED(right) FOR ARGENTINA HERE(down) COUNTRY / LONG-TIME-AGO TO-MAKE-INDEPENDENT(perf.) AMÉRICA DH:ALL NDH: AMERICA MAKE-INDEPENDENT(perf.) SAME WAR(left) WIN(from left to center) / WHO / WELL MEAN POINT(right) S-A-N M-A-R-T-I-N POINT(right) J-O-S-E PERSON(right) VERY-FAMOUS POINT(right) / POINT(right) TO-BE-TRUE LIVE(left) CORRIENTES PLACE(left) SMALL-TOWN(left) Y-A-P-E-Y-U SMALL-TOWN(left.hold NDH) POINT(point to NDH) FAMILY(left) POINT(left) BORN ALREADY BORN HERE(down) ARGENTINA BORN GROW(hold NDH) YEARS-OLD POINT(toward NDH) MORE-OR-LESS EARLY WHAT / PLACE ARGENTINA HERE(down) THERE-IS-NOT STUDY(left) / PROGRESS(left) THERE-IS-NOT / PLACE(left) MEAN FIELD(left) MOSTLY INDIANS HARVEST(left) MANY-THINGS(left) FIELD(left) / PEOPLE(left.pl) PARTICIPATE(left) MILITARY WHO / SPAIN POINT(up-right) COME(from up-right to down-left) LONG-TIME-AGO (up-right)CONQUER(down-left) / FAMILY COME-TO-LIVE(left)++ / [POINT(right) SAN-MARTIN BORN HERE(down) / FATHER MOTHER FAMILY SPAIN POINT(up.right)+]-BODYright [COME(left) COME-TO-LIVE(left) MONEY WEALTH COME-TO-LIVE(left) HARVEST(left) PLACE(left) INDIANS]-BODYleft |
| *I am going to tell you about a very famous person, who is highly renowned in Argentina. A long time ago he won the independence of all America, by winning many battles. This famous person is José de San Martín*. *He lived in a small town called Yapeyú, in the province of Corrientes [Argentina]. He was born and spent his early years here, in Argentina. When he was very young, his family realized that here there was no opportunity for him to study or progress, because there was mostly fieldwork. People used to join the Spanish army, since a long time ago these lands were conquered by Spain. Unlike San Martín, his family was born in Spain. They were a wealthy family, so they were able to come and live in the countryside, where they harvested lands of the native communities.* |

| **G) The two battling sides fragment (Video 5) / Timecode: 4:45 – 5:35** |
| --- |
| THINK FEEL ENGLAND GO-TO(future) CONQUER(left) / NO / BEFORE PRO1 MUST PREPARE FINE DECIDE GO-TO ENGLAND POINT(up.right) SHIP(up.right) GO-STRAIGHT(from up.right to down.left) PLACE(left) AMÉRICA(left) SOUTH ARGENTINA HERE COUNTRY GO-WITH-SHIP(from up.right to down.left) POINT(up.right) SPAIN DID-NOT-KNOW WHAT / SUSPECT(up-right) PRO1 GO-WITH-SHIP(from up.right to down.left) PRO1 ARRIVE(down.left) POINT(down.left) TIME THINK PRO1 PLAN PRO1 CONTRAVENE PRO1 COME-TO-LIVE(left) PRO1 INFORM(left) PLACE(left) CREATE POSS1 PLACE(left) / POINT(right) SAN-MARTÍN THINK KNOW POINT(left)+ ALREADY BATTLE(continuative.left)(2H) / AREA(left) WHO BATTLE(left)(2H) WHO / PERSON(left) SPAIN OTHER(leftmost) SPAIN [DH: SPAIN+]-BODY movement sideways (disjunctive) / BATTLE(left)(2H) (hold with NDH) / SPAIN+ WHY / NO SPAIN PLACE(left.center) WANT CONTINUE(left) RESPECT(up.right) KING OTHER(leftmost) BATTLE(leftmost)(1H) AGAINST(leftmost) KING KICK-OUT(left to right) WANT POINT(leftmost) WHO FIND(perf.left) BELGRANO POINT+(leftmost) BELGRANO FIND(leftmost) POINT(leftmost) // SAN-MARTÍN THINK WE-TWO(self.left) AGREE(left) WELL POINT(left). |
| *Knowing the English plans and taking advantage of the Spanish uncertainty of his whereabouts, San Martín decided to go straight from England to South America by sea. He arrived in Argentina planning on creating his own regiment. San Martín knew that [in Argentina] two sides have been battling for a long time. Who were these sides? Where they both from Spain? No, one of them wanted the King of Spain to continue ruling, whereas the other was against the King, and wanted to remove the Spanish Crown from power. In the latter, San Martín found Belgrano. San Martín realized that they both shared views.* |
